# Novel control of cardiac myofilament response to calcium by S-glutathionylation at specific sites of myosin binding protein C

**DOI:** 10.3389/fphys.2013.00336

**Published:** 2013-11-20

**Authors:** Bindiya G. Patel, Tanganyika Wilder, R. John Solaro

**Affiliations:** Department of Physiology and Biophysics and Center for Cardiovascular Research, College of Medicine, University of Illinois at ChicagoChicago, IL USA

**Keywords:** oxidative stress, sarcomeres, C-protein, cardiac relaxation

## Abstract

Our previous studies demonstrated a relation between glutathionylation of cardiac myosin binding protein C (cMyBP-C) and diastolic dysfunction in a hypertensive mouse model stressed by treatment with salt, deoxycorticosterone acetate, and unilateral nephrectomy. Although these results strongly indicated an important role for S-glutathionylation of myosin binding protein C as a modifier of myofilament function, indirect effects of other post-translational modifications may have occurred. Moreover, we did not determine the sites of thiol modification by glutathionylation. To address these issues, we developed an *in vitro* method to mimic the *in situ* S-glutathionylation of myofilament proteins and determined direct functional effects and sites of oxidative modification employing Western blotting and mass spectrometry. We induced glutathionylation *in vitro* by treatment of isolated myofibrils and detergent extracted fiber bundles (skinned fibers) with oxidized glutathione (GSSG). Immuno-blotting results revealed increased glutathionylation with GSSG treatment of a protein band around 140 kDa. Using tandem mass spectrometry, we identified the 140 kDa band as cMyBP-C and determined the sites of glutathionylation to be at cysteines 655, 479, and 627. Determination of the relation between Ca^2+^-activation of myofibrillar acto-myosin ATPase rate demonstrated an increased Ca^2+^-sensitivity induced by the S-glutathionylation. Force generating skinned fiber bundles also showed an increase in Ca-sensitivity when treated with oxidized glutathione, which was reversed with the reducing agent, dithiothreitol (DTT). Our data demonstrate that a specific and direct effect of S-glutathionylation of myosin binding protein C is a significant increase in myofilament Ca^2+^-sensitivity. Our data also provide new insights into the functional significance of oxidative modification of myosin binding protein C and the potential role of domains not previously considered to be functionally significant as controllers of myofilament Ca^2+^-responsiveness and dynamics.

## Introduction

In experiments reported here, we tested the hypothesis that oxidative modification of cardiac myosin binding protein C (cMyBP-C) at specific sites modifies myofilament response to Ca^2+^. cMyBP-C is a thick filament associated protein composed of eight IgI-like (C0–C5, C8, C10), three fibronectin-3-like domains (C6, C7, C9), region between C0 and C1 rich in proline and alanine residues (Pro-Ala-rich linker), and cardiac specific sequence (M-domain) that links C1 and C2 domains (Sadayappan and de Tombe, 2012). There is also a cardiac specific domain housed in the C5 domain. Gain and loss of function studies have shown that cMyBP-C is an important regulator of cardiac dynamics (Tong et al., 2008). cMyBP-C interacts with myosin S-2 (Harris et al., 2004; Ratti et al., 2011; Pfuhl and Gautel, 2012) and with titin (Sadayappan and de Tombe, 2012) and is believed to control the radial disposition of cross-bridges in relation to the thick filament proper (Colson et al., 2012a; Sadayappan, 2012; Sadayappan and de Tombe, 2012). For example, phosphorylation of the M-domain moves cross-bridges closer to the thin filament and promotes cross-bridge cycling kinetics (Colson et al., 2012b). There is also evidence of a direct interaction of MyBP-C with the thin filament, but the significance and presence of this reaction remains controversial and poorly understood (Harris et al., 2004; Kensler et al., 2011). Linkage of prevalent and penetrant mutations to familial cardiomyopathies points to the significance of cMyBP-C in homeostatic control of cardiac function (Harris et al., 2011; McNally et al., 2013).

In previous studies, we reported data indicating a new role for cMyBP-C in the response of the myocardium to hypertensive stress in a mouse DOCA-salt model stressed by administration of salt, deoxycorticosterone acetate and unilateral nephrectomy (Lovelock et al., 2012; Jeong et al., 2013). These mice demonstrated oxidative stress and a diastolic abnormality in hearts and isolated myocytes, which occurred with no apparent change in cellular Ca^2+^-fluxes (Lovelock et al., 2012). These findings indicate that altered Ca^2+^-responsiveness of the myofilaments might be involved, and indeed our experiments demonstrated enhanced myofilament response to Ca^2+^- with slowing of cross-bridge kinetics (Lovelock et al., 2012). We also reported a correlation of levels of MyBP-C S-glutathionylation with diastolic dysfunction with reversal of the oxidative stress by treating the DOCA-salt mice with tetrahydro-biopterin (Jeong et al., 2013). We identified S-glutathionylation of cMyBP-C as a post-translational modification likely to induce the altered response to Ca^2+^ (Lovelock et al., 2012; Jeong et al., 2013). Yet, we also found that myofilaments from the DOCA-salt model had significantly depressed levels of MyBP-C phosphorylation at Ser 282, which correlated with a depression of cardiac TnI (cTnI) phosphorylation at Ser 23, Ser 24. Thus, questions remained regarding whether the functional effects of MyBP-C could be demonstrated by direct glutathionylation of cMyBP-C at the same levels of phosphorylation of cMyBP-C and cTnI. A question also remained regarding the sites of the glutathionylation on cMyBP-C.

In the present experiments, we pursued an approach to these questions by developing *in vitro* conditions for direct S-glutathionylation of sarcomeric proteins. This approach provided a direct test of the hypothesis that this post-translational modification is the major mechanism of the altered sarcomeric response to Ca^2+^. Our findings support this hypothesis and also show for the first time that S-glutathionylation occurs on Cys residues in domains of cMyBP-C not generally expected to be major players in controlling myofilament function.

## Materials and methods

### Isolation of cardiac myofibrils and measurements of ATPase activity

Four month old female FVBN mice were deeply anesthetized with 60 mg/kg pentobarbital. The heart was quickly excised and rinsed in cold 0.9% sodium chloride. All methods were approved by the University of Illinois at Chicago Animal Care and Use Committee. We isolated myofibrillar fractions from ~50 mg wet weight of left ventricular tissue using a modification of procedures described by Solaro et al. (1971) and Layland et al. (2005). Membranes in the tissue were extracted by two homogenizations in 1 ml of a standard buffer with Triton X-100 (75 mM KCl, 10 mM imidazole, pH 7.2, 2 mM MgCl2, 2 mM EGTA, 1 mM NaN3, and 1% v/v Triton X-100) using a 2 ml Dounce homogenizer. Following centrifugation, pellets were washed twice with 1 ml standard buffer without Triton X-100 and resuspended in the assay buffer (A-70 containing 70 mM NaCl, 10 mM MgCl2, and 40 mM MOPS, pH 7.0) (Kobayashi and Solaro, 2006). A DC assay (Bio-Rad) was performed to determine protein concentration of the sample. Modifications of assays of myofibrillar activity were carried out on fresh isolated preparations. For *in vitro* glutathionylation (Chen et al., 2007), myofibrillar protein suspensions (0.2 mg/ml) were incubated for 1 h at room temperature in either A-70 buffer or A-70 containing various concentrations of oxidized glutathione (GSSG). Following the glutathionylation reaction, the myofibrils were suspended in an assay buffer containing 0.1 mg/ml protein, 35 mM NaCl, 5 mM MgCl_2_, 1 mM EGTA, 20 mM MOPS, pH 7.0 with CaCl_2_ to achieve a range of pCa (−log [Ca^2+^] values from pCa 7.8 to pCa. 4.6). Free Ca^2+^ concentration was calculated using WEBMAXC STANDARD. We determined myofibrillar ATPase activity at 30°C by starting the reaction with 1 mM ATP and stopping the progress by addition of trichloroacetic acid every 3 min for 15 min, during which Pi generation, as determined with a malachite green based assay, was linear (Kodama et al., 1986). Blank assays without protein did not demonstrate non-enzymatic ATP hydrolysis. Data were normalized to maximum activity. Graph Pad Prism 5.00 was used to analyze ATPase rates and to fit the data to the Hill equation to generate half maximally activating pCa values (pCa_50_) and Hill *n* values.

### Force measurement of skinned fiber bundles

Measurements of the force- Ca^2+^ relationship were carried out on fiber bundles from left ventricular papillary muscle of adult mice essentially as previously described (Evans et al., 2000). Female mice 4 months old were anesthetized as above and hearts were quickly excised and placed in ice cold high relaxing (HR) solution pCa 10.0 of the following composition in mM: K-propionic acid 41.89, MgCl_2_ 6.57, BES 100, EGTA 10, ATP 6.25, phosphocreatine 10, Na-azide 5, pH adjusted to 7.0 using KOH. The ionic strength of all solutions was 150 mM. All solutions contained protease inhibitors pepstatin (2.5 μg/ml), leupeptin (1 ug/ml) and phenylmethylsulphonyl fluoride (PMSF, 50 μm). Fiber bundles (150–200 μm in width and ~4–5 mM long) were dissected from papillary muscles. Membranes were extracted from the fiber bundles by immersing them for 30 min in HR buffer containing 1% Triton X-100. We mounted the fiber bundles between a force transducer and micromanipulator, and, after an initial contraction to maximum force and return to relaxation, we set the sarcomere length at 2.2 μm using laser diffraction patterns. A, The fibers were then exposed to solutions of incrementally increasing Ca^2+^ concentrations ranging from 10^−7^ to 10^−4.5^ M and force was recorded to determine the force-pCa relations. Fibers were then incubated in 5 mM GSSG in A-70 as prepared above for 10 min, and then subjected to the varying Ca^2+^ concentrations. This process was repeated using 10 mM dithiothreitol (DTT) solubilized in A-70 for 10 min. No changes in force or Ca-sensitivity were observed when we treated control skinned fibers (no BSSG) with DTT. Tension was calculated by dividing the force by the cross-sectional area, as described previously (Evans et al., 2000). Assuming a cylindrical shape we determined radius from measurements of two perpendicular planes at three points along the fiber. The mean radius was used to calculate the cross-sectional area. Data from each experimental run were fit to the Hill equation with pCa as the independent variable for non-linear regression using Graphpad Prism 5 software. The results were than averaged for reporting in the figures.

### Immunoblotting

Control and GSSG treated myofibrils in A-70 assay buffer were solubilized in a non-reducing 2X Laemmli buffer (Laemmli, 1970) (4% SDS, 20% glycerol, 0.004% bromophenol blue, and 0.125 M Tris HCl pH 6.8) with 25 mM N-ethylmaleimide (NEM) in a 1:1 ratio. A negative control was prepared by adding 10 mM DTT to myofibril proteins. 20 μ g of total protein was applied to 1D 12% non-reducing resolving SDS-PAGE gel (Fritz et al., 1989) and transferred onto a 0.2 μM PVDF membrane (Matsudaira, 1987). The blot was blocked in 5% non-fat dry milk with 2.5 mM NEM for 1 h. Anti-glutathione mouse monoclonal primary antibody (Virogen) was used at 1:1000 dilution along with anti-mouse HRP-conjugated secondary antibody (Sigma) at 1:40,000 dilution to detect for S-glutathionylation (Hill et al., 2010). Skinned fibers (4–6 fiber bundles) from force measurement studies were solubilized in 30 μl of the 2X Laemmli buffer with 25 mM NEM. Proteins in the samples (4–10 μl) were separated on 1D 12% non-reducing resolving SDS-PAGE gel. The SDS gels showed the same pattern of proteins and loading as previously reported (12; Figure 5). Transfer and western blot procedure was same as above. Optical density of the bands was measured with ImageQuant TL (GE Healthcare) and exported to Excel for statistical analysis.

### Mass spectrometry

An 8% non-reducing 1D SDS-PAGE gel was stained with Imperial Protein Stain (Thermo) according to manufacturer's protocol. The band around 140 kDa (MyBP-C) was cut from the gel and subjected to in-gel digest with Trypsin Gold (Promega). Reduction and alkylation steps were omitted to preserve the S-glutathionylation of the proteins. Pooled digestion extracts were concentrated via Speed Vac to less than 20 μl and brought up to 40 μl with mobile phase solution (5% ACN and 0.1% formic acid). Peptides were filtered with 0.22 μm PVDF Millipore Ultrafree-MC spin filter and 35 μl of sample was analyzed with Thermo Finnigan LTQ hybrid linear ion trap—Fourier Transform ICR mass spectrometer coupled to Dionex U3000 nano LC. Dionex acclaim PepMap100 C18 trapping column (500 μm × 5 mm column packed with 5 μm, 100 A Symmetry C18 material) was used to concentrate the samples at flow rate of 50 μl/min and Agilent Zorbax 300SB-C18 Nanoflow column (75 μm × 150 mm column packed with 3.5 μm, C18 material) was used for separation at flow rate of 0.250 μl/min. Peptides were eluted with a linear gradient of 5–45% solution B (95% ACN, 0.1% formic acid) through New Objective uncoated SilicaTips™ (5 cm long, tubing OD 360/75 μm, tip ID 8 μm). Peptides were ionized via electrospray ionization with LTQ source voltage set to 1.0 kV and capillary temperature set to 200°C. Mass spectra were obtained in positive ion mode over m/z range of 400–1800 at a resolution of 50,000 and top ten most intense ions were selected for tandem MS. MS/MS were obtained in collision induced dissociation mode with minimum signal required 5000, isolation width 3, and normalized collision energy 35.

Mass spectrometry data were collected with Xcalibur 2.0.7 software as.Raw file, which were converted to mzXML and MGF files using MassMatrix MS Data File Conversion tool (Xu and Freitas, 2009). Mascot search engine was used to analyze the MS/MS data (Perkins et al., 1999). Searches were performed using NCBInr Mus musculus and a decoy (automatically generated reversed protein sequences) database with peptide tolerance of ±15 ppm. Variable modifications were set to glutathione (C). The results are representative of three similar and separate mass spectrometry runs.

### Statistical analysis

We determined pCa values at half-maximum ATPase activity and force generation from data normalized to maximum activity. The data were fit to a modified Hill equation (Takeda et al., 1997; Kobayashi and Solaro, 2006). Statistical significance was determined using paired Student *t*-test or One Way ANOVA followed by Newman-Keuls test where appropriate. Data are presented as means ± SEM with significance set at *p* < 0.05.

## Results

### Myofibrilar ATPase activity

**Figure 1:**
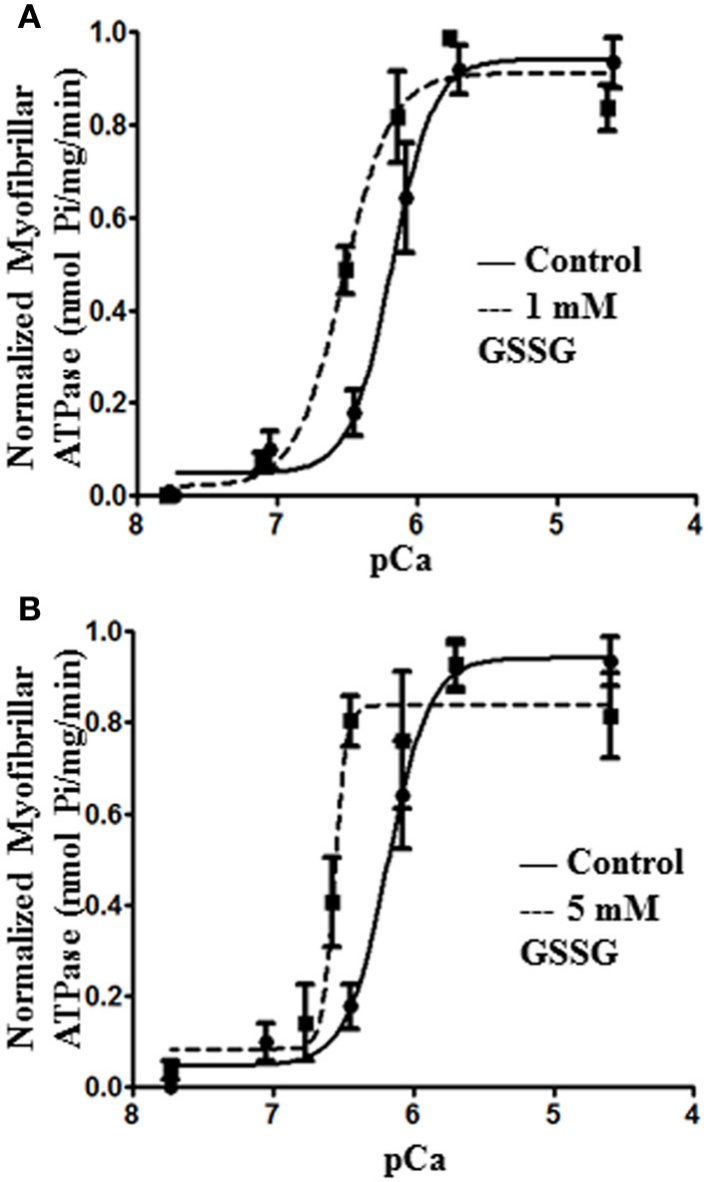
**Myofibril pCa-ATPase activity of control and GSSG treated samples. (A)** *In vitro* ATPase activity of myofibrils prepared from wild type FVBN mouse heart treated with 1 mM GSSG for 1 h. pCa50 for control samples is 6.17 ± 0.09 (shown also in panel **B**) and for 1 mM GSSG treated samples is 6.50 ± 0.07 **(B)** *In vitro* ATPase activity of myofibrils prepared from wild type FVBN mouse heart treated with 5 mM GSSG for 1 h. pCa50 for control samples is 6.17 ± 0.09 and for 5 mM GSSG treated samples is 6.60 ± 0.03. Data are expressed as mean ± standard error of mean, *n* = 4 from 1 preparation. In both panels **(A)** and **(B)** the change in pCa50 was significant (*P* < 0.05). *n*_H_, minimum pCa, and maximum pCa values were not significantly different.

In order to determine direct effects of glutathionylation on myofilament response to calcium, we compared ATPase activities of isolated myofibrillar preparations before and after treatment with 1 and 5 mM GSSG (Figure 1). Compared to controls, myofibrils treated with either 1 mM (Figure 1A) or 5 mM GSSG (Figure 1B) demonstrated a significant leftward shift in the relation between pCa and myofibrillar ATPase activity. In the case of modification by 1 mM GSSG, the half- maximally activating pCa value (pCa_50_) was 6.50 ± 0.07 (*n* = 4) compared to a pCa_50_ of 6.17 ± 0.09 (*n* = 4) for controls. With 5 mM GSSG the pCa_50_ was 6.60 ± 0.03 (*n* = 4) compared to controls with pCa50 of 6.02 ± 0.07 (*n* = 4). The Hill coefficients, minimum, and maximum ATPase rates did not change significantly between the control myofibrils and myofibrils treated with either 1 or 5 mM GSSG.

### Glutathionylation of myofibrillar preparations

**Figure 2:**
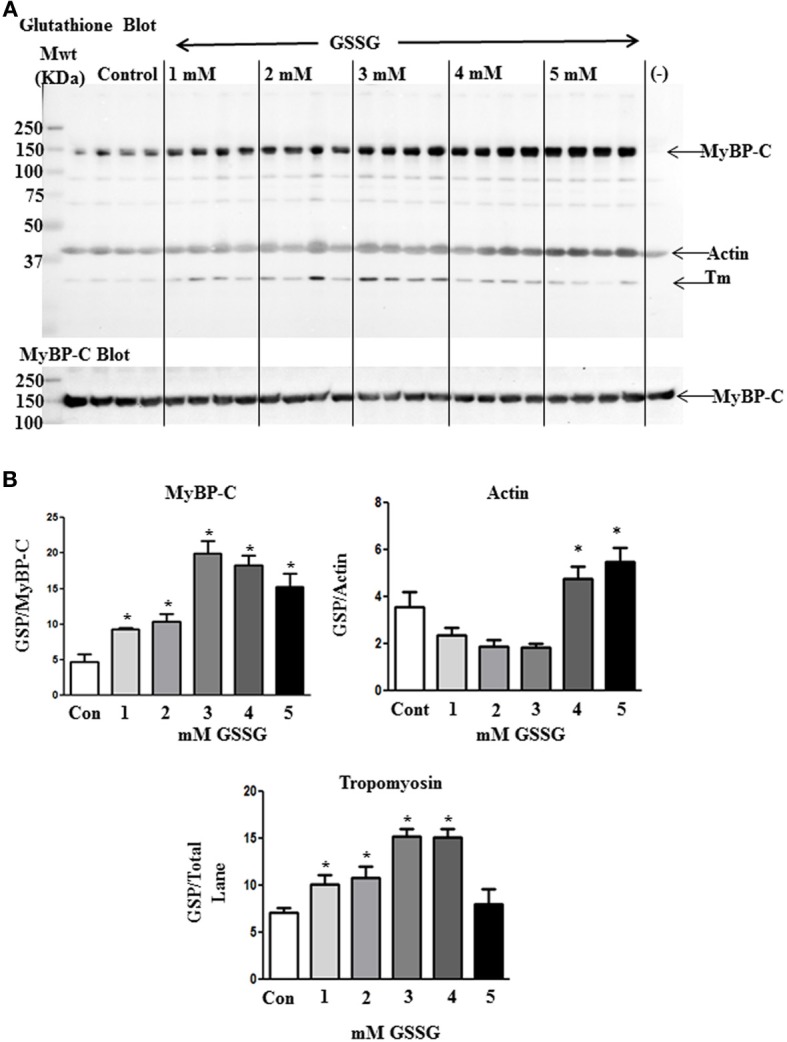
**Western blot analysis of proteins subject to glutathionylation during ATPase assay.(A)** Control myofibrils and myofibrils treated with increasing concentrations of GSSG (20 μg) were probed with anti-GSH (Virogen) antibody to detect glutathionylated proteins. (−) lane contains myofibrils treated with 10 mM DTT after 5 mM GSSG treatment to serve as a negative control. The blot was also probed with myosin binding protein C antibody for identification of glutathione band. **(B)** Load normalized quantitative results in arbitrary units showing significant difference in glutathionylation (GSP) of MyBP-C between non-treated and GSSG treated samples. Data are also shown for actin and tropomyosin bands. Data are expressed as mean ± standard error of mean, *n* = 4 (^*^P ≤ 0.05).

To determine the mechanism for the higher Ca^2+^, sensitivity of ATPase rate for the GSSG treated myofilaments, we analyzed the samples by Western blot analysis. Figure 2A shows blots for control samples and samples treated with increasing concentrations of GSSG. The Western blots revealed increased glutathionylation of a band identified as MyBP-C by probing with a specific antibody. As summarized in Figure 2B, all samples treated with GSSG demonstrated significant increases in glutathionylation compared to controls. Samples treated with 3, 4, and 5 mM GSSG had significantly increased levels of MyBP-C glutathionylation compared with the 1 mM GSSG treated samples, and the samples treated with 3 and 4 mM GSSG had significantly higher levels of glutathionylation than samples treated with 2 mM GSSG. Levels of glutathionylation were not significantly different among the 3, 4, and 5 mM GSSG treated groups. Western blots also detected glutathionylation of actin, but there were no significant differences between the GSSG treated groups and the control although the levels at 4 and 5 mM GSSG treatment were higher than those at 1, 2, and 3 mM GSSG treatment. Another band with mobility faster than that of actin and likely to be tropomyosin showed glutathionylation. However, the glutathionylation detected in this band did not show a dependence on dose of GSSG, and was the same as the control at 5 mM GSSG treatment.

### The effect of GSSG and DTT on skinned fiber force generation

**Figure 3:**
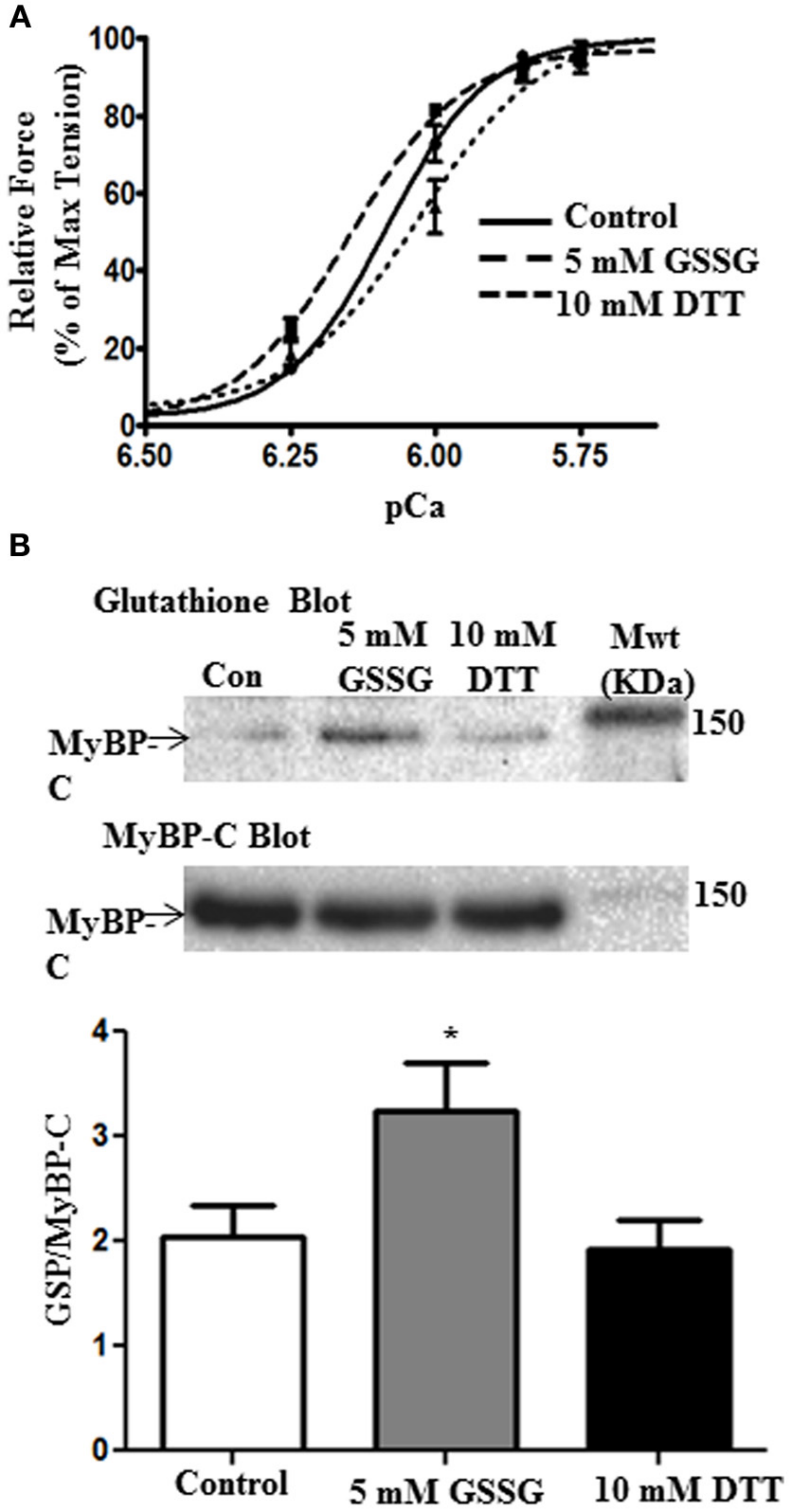
**The effects of GSSG and DTT on force generating parameters of skinned papillary fibers. (A)** Data represent the force-pCa relationship in skinned cardiac muscle fibers recorded primarily using only the varying increasing Ca^2+^ solutions, then followed by treatment with 5 mM GSSG in A-70 for 10 min and subsequently bathed in the Ca^2+^ solutions. The fibers were then incubated in 10 mM DTT in A-70 for 10 min and were then bathed in increasing Ca^2+^ solutions again. The pCa50 values were all statistically significant between treatments. pCa50 values for control fibers, 5 mM GSSG treatment, and 10 mM DTT treatment were 6.09 ± 0.01, 6.15 ± 0.01, and 6.03 ± 0.02, respectively. Data represented as mean ± SEM, *n* = 4 for all groups, significance was set at *p* < 0.05. (*n*_H_, minimum pCa, and maximum pCa values were not significant). **(B)** Western blot analysis of skinned fiber showing significant difference in glutathionylation levels between control and GSSG treated fibers (*p* = 0.05). Glutathionylation levels are also significantly decreased when fibers were treated with DTT compared to the GSSG treated fibers (*p* = 0.03). There is no significant difference between control and DTT treatment. Data are expressed as mean ± standard error of mean, *n* = 6.7 (^*^P ≤ 0.05).

We also determined the effect of glutathionylation on the force-pCa relation of skinned fiber bundles. The fiber bundles were first incubated for 10 min with A-70 buffer alone or 5 mM GSSG in A-70 buffer and then switched to HR for determination of force at various pCa values. As illustrated in Figure 3, skinned fiber Ca^2+^ sensitivity was significantly higher after GSSG incubation as indicated by pCa_50_ of 6.15 ± 0.01 (*n* = 4) compared to controls with pCa_50_ of 6.09 ± 0.01 (*n* = 4). This increase in pCa_50_ with direct glutathionylation is similar the increase in pCa_50_ we reported when comparing myofilaments sham mice and DOCA/salt mice [Figure 4A in Jeong et al. (2013)]To determine the reversibility of the effect of GSSG, we measured the force-pCa relation in a protocol in which we incubated the fiber bundles in 5 mM GSSG for 10 min followed by 10 mM treatment with DTT. As shown in Figure 3A the increased Ca^2+^ sensitivity associated with GSSG treatment was reversed after incubation in DTT as indicated by pCa_50_ of 6.03 ± 0.02 (*n* = 4). Cooperativity of activation, as measured by the Hill slope tended to decrease after GSSG incubation and return to control levels after DTT incubation, but none of the changes were statistically significant. As with the myofibrillar preparations, Western blots of the skinned fiber preparations revealed glutathionylation of MyBP-C (Figure 3B). The 5 mM GSSG treated fibers showed higher glutathionylation level than control fibers, and the level of glutathionylation returned to control levels following 10 mM DTT treatment. Although we detected glutathionylation of myosin heavy chain and actin in the skinned fibers treated with GSSG (data not shown), but there were no significant changes in glutathionylation level between the three groups for both proteins.

### Mass spectrometry

**Figure 4:**
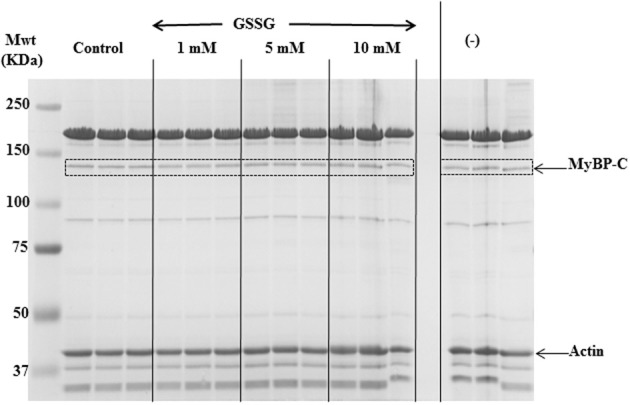
**SDS-PAGE gel for in-gel digestion.** 8% SDS-PAGE gel stained with mass spectrometry compatible Imperial Protein Stain (Thermo); bands at ~140 kDa (dotted boxes) were cut out for mass spec analysis.

**Figure 5:**
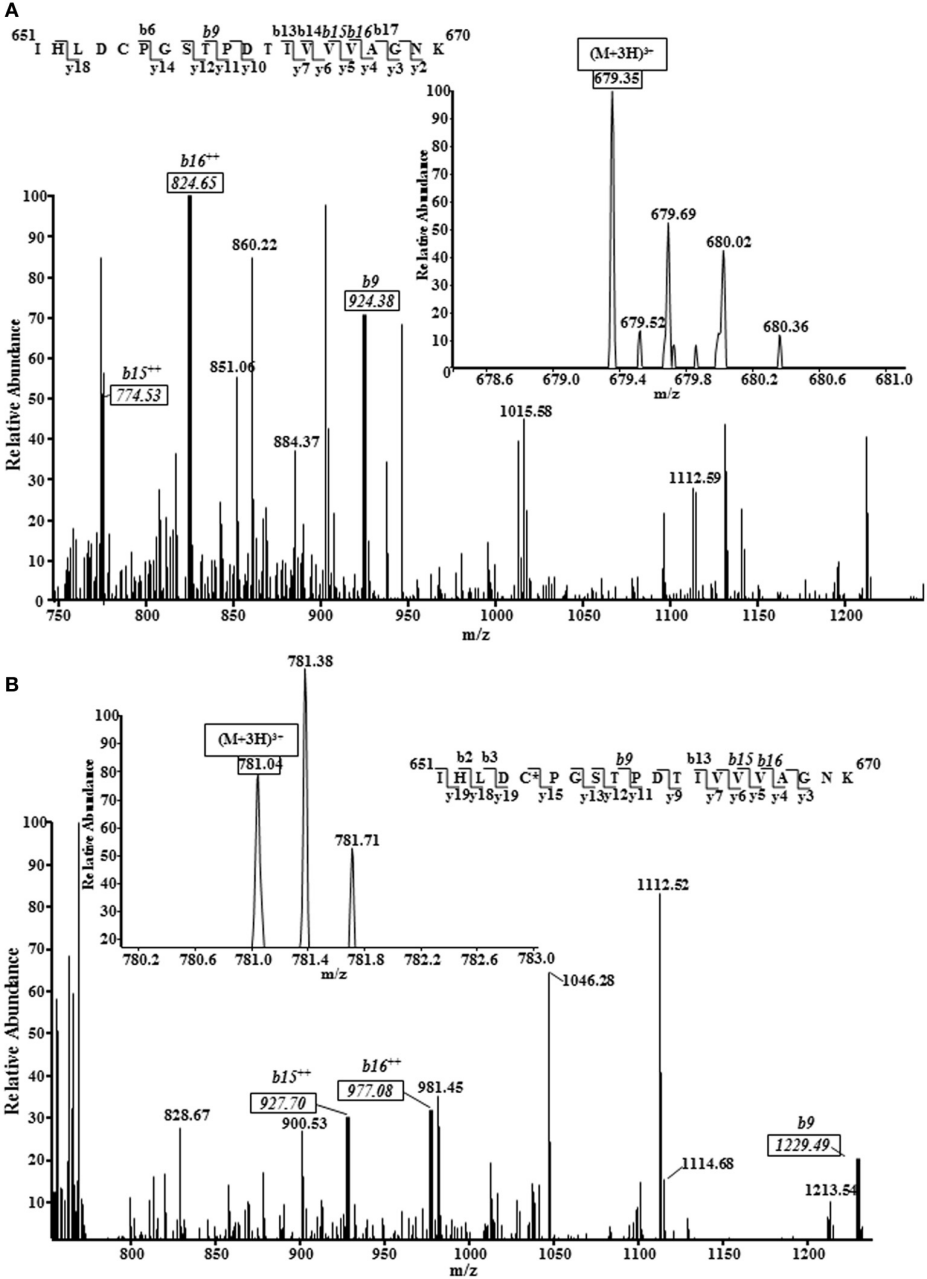
**Representative mass spectra (MS) and tandem mass spectra (MS/MS) of a triply protonated cMyBP-C peptide (^651^IHLDCPGSTPDTIVVVAGNK^670^) showing Cys^655^ glutathionylation.** 1 mM GSSG treated myofibrils subjected to mass spectrometry analysis revealed cysteine 655 of the C5 domain of mouse cardiac myosin binding protein C as a target for S-glutathionylation. The same site, along with 2 other sites, was also identified in 5 mM GSSG treated myofibrils. **(A)**, MS/MS of unmodified peptide showing several key b ions (solid boxes); inset figure shows the precursor ion (m/z 679.35^3+^) mass spectrum. **(B)**, MS/MS of glutathionylated peptide (GSH covalently bound to amino acid residue identified by asterisk) showing the same b ions (solid boxes) marked in the unmodified peptide spectrum shifted by 305 Da (singly charged b ions) or 152.5 Da (doubly charged b ions). Inset figure shows the modified precursor ion (m/z 781.04^3+^) mass spectrum. *Note:* The peptide sequences in both figures demonstrate all the b and y ions that were discovered; MS/MS spectrum shown is scaled (~750–1250 m/z) to show key ions.

We employed tandem mass spectrometry to identify glutathionylated proteins and determine the sites of cMyBP-C glutathionylation. Mass spectra resulting from the LC/MS/MS of the ~140 kDa band (Figure 4) identified myosin-binding protein C, cardiac-type (gi|134031947), with 43–50% sequence coverage as the number one hit. Tryptic peptides of cMyBP-C were analyzed for a mass shift of 305 Da, indicating covalent attachment of GSH to one of the peptide residues. Three separate peptides, ^651^IHLD**C**PGSTPDTIVVVAGNK^670^, ^475^VEFE**C**EVSEEGAQVK^489^, and ^605^LTIDDVTPADEADYSFVPEGFA**C**NLSAK^632^, were found to be glutathionylated (Figure 5, Table 1). When the myofibrils were treated with 1 mM GSSG, only peptide ^651^IHLD**C**PGSTPDTIVVVAGNK^670^ was found to be glutathionylated at Cys^655^. The precursor ion for the triply charged glutathionylated peptide was observed at m/z 781.04^3+^ compared to the unmodified precursor peptide observed at m/z 679.35^3+^. Comparison between select b and y product ions of the glutathionylated and unmodified peptide (Figures 5A,B) show mass shift of 305 Da (singly charged ions) or 152.5 Da (doubly charged ions). With treatment of 5 mM GSSG, we identified all three of the above peptides to be glutathionylated at Cys^655^, Cys^479^, and Cys^627^. The triply charged glutathionylated peptide ^475^VEFE**C**EVSEEGAQVK^489^ was observed at m/z 663.28^3+^ and ^605^lTIDDVTPADEADYSFVPEGFA**C**NLSAK^632^ glutathionylated precursor peptide was observed at m/z 1098.49^3+^. Expect value of less than 0.1 was considered to be significant identification of each individual peptide. Significant identification of glutathionylated cMyBP-C peptides was not made in the control non-treated myofibril samples.

**Table 1:** **Significantly glutathionylated peptides discovered by mass spectrometry**.

**GSSG (mM)**	**Accession number**	**Description**	**Score**	**Percent coverage**	**Glutathionylated peptide**	**Charge**	**Observed mass**	**Peptide mass tolerance (ppm)**	**Miss cleavages**	**Ions score**	**Expect**
1	gi|134031947	myosin-binding protein C, cardiac-type [Mus musculus]	7820	42.9	K.IHLDC*PGST PDTIVVVAGNK.L	3+	781.04	−5	0	41	0.0033
5	gi|134031947	myosin-binding protein C, cardiac-type [Mus musculus]	8033	49.7	K.IHLDC*PGST PDTIVVVAGNK.L	3+	781.04	−5	0	51	0.00033
					K.IHLDC*PGST PDTIVVVAGNK.L	3+	781.04	−5	0	48	0.00071
					K.IHLDC*PGST PDTIVVVAGNK.L	3+	781.04	−2	0	51	0.00041
					K.IHLDC*PGST PDTIVVVAGNK.L	3+	781.04	−2	0	50	0.00049
					R.VEFEC*EV SEEGAQVK.W	3+	663.28	0	0	45	0.00066
					R.VEFEC*EV SEEGAQVK.W	3+	663.28	0	0	36	0.0051
					R.VEFEC*EV SEEGAQVK.W	3+	663.28	1	0	30	0.018
					K.LTIDDVTPADE ADYSFVPEGFAC *NLSAK.L	3+	1098.49	5	0	40	0.0034
					K.LTIDDVTPADE ADYSFVPEGFAC *NLSAK.L	3+	1098.49	3	0	40	0.0031

Myofibrils treated with 1 mM and 5 mM GSSG. Mass spectrometry identified three separate sites as targets for S-glutathionylation. Note the increase in amount of peptides discovered to be glutathionylated and new sites for glutathionylation when myofibrils are treated with increased amount of GSSG.

## Discussion

**Figure 6:**
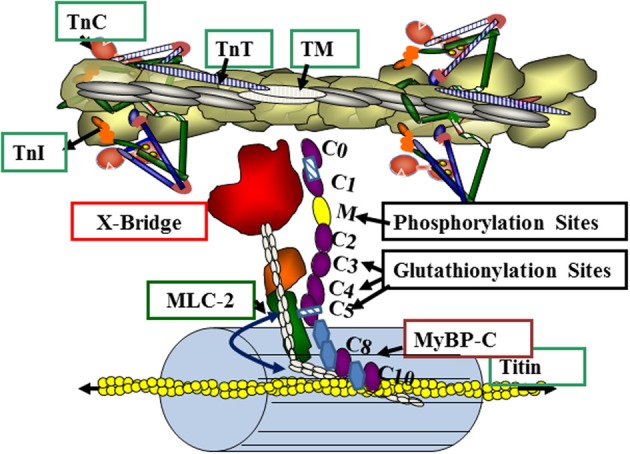
**Myosin binding protein C (MyBP-C) interactions in the overlap region of the sarcomere.** The illustration depicts a relaxed state with troponins (TnC, TnT, TnI) and tropomyosin (TM) blocking the actin-cross-bridge reaction. Myosin light chain 2 (MLC2) and MyBP-C contribute to radial movements of the myosin heads relative to the thick filament backbone (as indicated by the double headed arrow). MyBP-C has interaction sites via the M domain with the neck region of myosin (S2) and with titin via C-terminal domains C8, C9 and C10. MyBP-C may also interact directly with the thin filament. See text and results for details.

Our data provide support for the hypothesis that S-glutathionylation of MyBP-C is functionally significant in controlling myofilament response to Ca^2+^. Figure 6 illustrates a current concept of the position of MyBP-C in the sarcomere with the potential for interactions with thin filaments, myosin, and titin. Our data indicate that the sites modified by S-glutathionylation occur at Cys^479^, Cys^627^, and Cys^655^, and are in the poorly understood C3, C4, and C5 domains of MyBP-C. These domains have been previously considered to be more important in the structure and assembly of MyBP-C but not with major importance in the mechanism of action of MyBP-C, in which focus has been on the CO, L, C1, and especially the cardiac specific phosphorylation sites in the M domain. Thus, in addition to phosphorylation, which has well documented effects on cardiac MyBP-C function (Cazorla et al., 2006; Tong et al., 2008; Sadayappan et al., 2009; Hill et al., 2010) redox related post-translational modifications need to be considered.

With the discovery that many proteins are targets for glutathionylation, the search for functional implications of this post-translational modification has taken on new significance (Brennan et al., 2006; Pastore and Piemonte, 2012). Brennan et al. (2006) briefly reported the presences of S-gluthionylation of MyBP-C in rat hearts, but functional implications were not assessed. Glutathionylation is a form of oxidation involving formation of a mixed disulfide between the tri-peptide glutathione (GSH) and a protein cysteine residue. The tri-peptide glutathione is the most abundant non-protein thiol present in cells, with concentrations varying from 1–10 mM depending on the cell type. For example, the amount of glutathione that is present in the heart is 5-fold lower than that found in the liver (Ishikawa and Sies, 1984). Glutathione has many functions including its role as an antioxidant, indicator of oxidative stress, and ROS scavenger (Pastore and Piemonte, 2012). Under physiological conditions, much of the cell's GSH pool is present in the reduced form and less than 1% is present in oxidized GSSG. Under oxidative stress, the ratio of GSH/GSSG decreases and promotes protein glutathionylation. When we added DTT to the skinned fiber preparations (Figure 3) there was a right shift of the pCa-force relation indicating the presence of redox modified sarcomeres in the controls. Although this indicates the possible glutathionylation of MyBP-C in the controls, *in situ* levels remain to be determined. GSSG reacts with protein thiols through a disulfide exchange mechanism to form a protein mixed disulfide. Cardiac myocytes provide an excellent cell type for investigation of the potential effects of protein S-glutathionylation in both physiological and pathological redox signaling. Results presented here provide further support that specificity for a particular protein and regulatory process and may form an important aspect of signaling via S-glutathionylation. In the case of the DOCA-salt mouse model of hypertension, we could find no change in the levels and dynamics of intracellular Ca^2+^ transients, even though it has been reported that glutathionylation of L-type Ca^2+^ channel results in an increase in Ca^2+^ influx and an increase in diastolic Ca^2+^ in cardiac myocytes (Tang et al., 2011). However, there is a reported glutathionylation of cardiac L-type Ca^2+^ channels associated with ischemic heart disease (Tang et al., 2011). Thus, it appears important to evaluate the role of post-translational modifications by glutathionylation in the context of the particular physiological or pathophysiological condition.

Although it is likely that other proteins in cardiac sarcomeres are S-glutathionylated, based on the present results and our previous data, the impact of S-glutathionylation of MyBP-C appears to be of relatively high significance in controlling cardiac relaxation and as an important mechanism for the diastolic dysfunction associated with hypertension induced oxidative stress. There have been reports that reversible glutathionylation of actin at Cys^374^ has functional impact on actin polymerization (Wang et al., 2001; Dalle-Donne et al., 2003) and actomyosin-S1 ATPase activity (Pizarro and Ogut, 2009). However, in the present study, we did not observe actin glutathionylation at Cys^374^. Moreover, we could detect no actin glutathionylation in cardiac myofilaments from the DOCA-salt model, although there was an increase in Ca^2+^ sensitivity compared to controls (Lovelock et al., 2012). Western blots (Figure 2B) showed a variable level of glutathionylation of actin and a protein migrating with the mobility of tropomyosin. However, unlike the case with MyBP-C, the level of glutathionylation of actin and tropomyosin was not correlated with the effects of GSSG on myofibrillar ATPase activity. We also did not detect glutathionylation of the reactive Cys^707^ of myosin sub-fragment 1 (S1) (Prochniewicz et al., 2008). These data support our conclusion that in some conditions glutathionylation of cMyBP-C is a dominant oxidative stress related post-translational mechanism for control of myofilament Ca^2+^sensitivity.

Our data indicate high susceptibility of cMyBP-C for modification by glutathionylation at specific cysteine residues, which may be exposed in the in the three dimensional structure for ease of access by GSSG. Glutathionylation has also been correlated with relatively low pKa values for susceptible Cys residues (Pastore and Piemonte, 2012). It would be of interest, therefore, to examine the position and comparative pKa values of the Cys^479^, Cys^627^, and Cys^655^. The modification in the C5 domain may be of particular interest inasmuch as this domain contains a cardiac specific region. Some evidence points to a functional significance of these C-terminal domains of cMyBP-C in the heart. Interactions of this region with titin and light meromyosin have been documented but not extensively analyzed in terms of functional significance (Yang et al., 1998; Sadayappan and de Tombe, 2012). Surprisingly the C-terminal regions outside C0–C4 bind to actin equally as well as the full length MyBP-C, and the speculation was made by Rybakova et al. (2011) that this actin-MyBP-C interaction may be relatively more specific than the relatively non-specific electrostatic interactions of the N-terminal regions. Missense mutations inducing familial hypertrophic cardiomyopathy occur in all of the MyBP-C domains (Flashman et al., 2004; Harris et al., 2011) indicating that each domain has a special significance in cardiac homeostasis or that effects of modifications are transmitted to others in the domain network. Studies by Palmer et al. (2011) have provided some indirect insights into a potential functional role of regions of MyBP-C outside the N-terminal domains and phosphorylation sites. Their studies indicated that phosphorylation and the presence of the N-terminal domains of MyBP-C provide structural support and radial rigidity to the myofilament lattice. However, the presence of cMyBP-C also provided a longitudinal rigidity in the myofilament lattice that did rely on phosphorylation of the N-terminus. We hypothesize regions glutathionylation of those regions may be significantly involved in maintenance of longitudinal rigidity as well as potential interactions with as well dwell time of cross-bridges in their reaction with thin filaments.

## Conclusion

In conclusion our data emphasize and support our earlier data indicating the potential for redox related post-translational modification of MyBP-C as a significant factor controlling cardiac function. Our results also emphasize the need for better understanding of the role of the C-terminal regions of MyBP-C that contain thiols highly reactive with GSSG. The specificity of the correlation of S-glutathionylation with myofilament function also indicates that effects of oxidative stress need to be considered in the context of the particular circumstances generating ROS. It will be important in further studies to determine interactions among these redox related post-translational modifications and phosphorylation. Whether the existence of a S-glutathionylated form has any relevance in a variety of cardiac disorders needs to be further explored. Our findings may also relate to the use of serum levels of MyBP-C as a biomarker for acute myocardial function (Jacquet et al., 2009; Sadayappan, 2012). We also acknowledge that the general significance of our findings must await demonstration of altered S-glutathionylation in other disorders of the heart.

## Author contributions

Bindiya G. Patel performed gel analysis, the ATPase measurements, and mass spectrometry. Tanganyika Wilder performed the force measurements and gel analysis. Bindiya G. Patel and Tanganyika Wilder also wrote drafts of the manuscript. R. John Solaro designed the experiments and supervised all aspects of the study and wrote the final draft with input from Bindiya G. Patel and Tanganyika Wilder.

### Conflict of interest statement

The authors declare that the research was conducted in the absence of any commercial or financial relationships that could be construed as a potential conflict of interest.
